# H9N2 influenza virus in China: a cause of concern

**DOI:** 10.1007/s13238-014-0111-7

**Published:** 2014-11-11

**Authors:** Yipeng Sun, Jinhua Liu

**Affiliations:** Key Laboratory of Animal Epidemiology and Zoonosis, Ministry of Agriculture, College of Veterinary Medicine, China Agricultural University, Beijing, 100083 China

**Keywords:** Influenza, Poultry, H9N2, Evolution, Virulence, Antigenic drift

## Abstract

The recent human infection with avian influenza virus revealed that H9N2 influenza virus is the gene donor for H7N9 and H10N8 viruses infecting humans. The crucial role of H9N2 viruses at the animal-human interface might be due to the wide host range, adaptation in both poultry and mammalian, and extensive gene reassortment. As the most prevalent subtype of influenza viruses in chickens in China, H9N2 also causes a great economic loss for the poultry industry, even under the long-term vaccination programs. The history, epidemiology, biological characteristics, and molecular determinants of H9N2 influenza virus are reviewed in this paper. The contribution of H9N2 genes, especially RNP genes, to the infection of humans needs to be investigated in the future.

## INTRODUCTION

H9N2 influenza virus has become endemic in different types of terrestrial poultry in multiple countries on the Eurasian continent, resulting in great economic losses due to reduced egg production or high mortality associated with co-infection with other pathogens (Lee and Song, 2013; Shanmuganatham et al., 2013; Sun et al., 2010b; Zhao et al., 2013). Epidemiological and genetic studies revealed that the hemagglutinin (HA) gene of the H9N2 influenza viruses could be divided into Eurasian avian and American avian lineages. The Eurasian avian lineage involved three distinct lineages, including A/chicken/Beijing/1/94-like (BJ/94-like), A/quail/Hong Kong/G1/97-like (G1-like), and A/duck/Hong Kong/Y439/97 (Y439-like). BJ/94-like and G1-like viruses have been prevalent mainly in China since the mid-1990s (Sun et al., 2010b).

## HISTORY AND EPIDEMIOLOGY OF H9N2 VIRUS IN AVIAN SPECIES IN CHINA

In China, which is regarded as an epicenter of avian influenza viruses, the H9N2 virus has been detected in multiple avian species, including chicken, duck, quail, pheasant, partridge, pigeon, silky chicken, chukar, and egret (Wang et al., 2012a; Xu et al., 2007).

The first outbreak of the H9N2 influenza virus in China occurred in Guangdong province of Southern China during November 1992 to May 1994; the outbreak affected, amongst others, seventeen chicken farms and two rare bird farms. These H9N2 viruses killed broilers with mortality of 10%–40%, and reduced the laying rates by 14%–75% (Chen et al., 1994; Zhang et al., 1994); however, they only caused mild flu-like symptoms in specified pathogen free (SPF) chickens in the laboratory (Chen et al., 1994). After this outbreak, the H9N2 infection sporadically occurred in chickens, ducks, and goose and spread to Northern China (Chen and Chen, 1999). The H9N2 influenza virus spread to most provinces of China within two months in 1998, and is now the most prevalent subtype of influenza viruses in chickens in China (Pan et al., 2012).

H9N2 infections occur throughout the whole year, with lower morbidity in the summer. The isolation rate of H9N2 influenza virus in apparently healthy chickens, ducks, and other minor poultry species (such as quail, partridges, chukar, pheasant, and guinea fowl) in live poultry markers between 2000 and 2005 were 2.5%, 0.18%, and 4.7%, respectively (Xu et al., 2007). The isolation rate of H9N2 virus in the poultry in live poultry markets in Shanghai province of China was 2.6% between 2008 and 2010 (Wang et al., 2014).

## CLINICAL SIGNS IN CHICKENS OF H9N2 VIRUS

The H9N2 influenza virus can be transmitted by air droplet, dust, feed, or water (Liu, 2012). On its own, the H9N2 influenza did not induce obvious clinical signs or deaths in chickens (Sun et al., 2010b). Chickens usually seemed to be healthy; few showed depression and ruffled feathers. Increased oral mucus was observed in the infected chickens (Sun et al., 2010b). At autopsy, local pulmonary consolidation and petechiae in throat, trachea, and/or intestine has been observed. The virus replicated itself efficiently in tracheas, while the inoculated chickens rarely shed detectable viruses in the cloacal swabs (Li et al., 2005).

H9N2 influenza virus makes chickens more susceptible to secondary infections, especially *Escherichia coli* infections with a mortality rate of at least 10%. In addition, the trachea or bronchi are easily embolized by mucus when the ventilation is poor, leading to severe respiratory disease and death. Chickens are immunized with H9N2 vaccines in China. Therefore, outbreak of H9N2 infections is common between vaccination. Commercial broilers are most commonly infected with H9N2 virus because most of them were very young and kept under poor conditions. About 60% of the poorly ventilated intensive broiler farms are infected by the H9N2 influenza virus. The morbidity of H9N2 infected laying hens is about 10%. If the laying hens were infected by H9N2 influenza during the laying period, the laying rate reduced to about 20%, and the birds were characterized by severe follicular congestion, and secretions are observed in fallopian tubes and uterus (Zhang, 2013).

## VACCINATION APPLICATION OF H9N2 VIRUS IN CHINA

To prevent H9N2 infection in chickens, China implemented long-term vaccination programs in chicken farms as early as 1998 (Li et al., 2005; Zhang et al., 2008). Most of the H9N2 vaccines are inactivated vaccines. At least over twenty different commercial vaccines are used in China, with the vaccines are frequently updated. However, H9N2 avian influenza viruses continues to persist in chicken populations, even in vaccinated flocks (Zhang et al., 2008).

## ANTIGENIC DRIFT OF H9N2 VIRUS IN CHINA

H9N2 viruses isolated from chickens in China underwent antigenic drift to evolve into distinct antigenic groups (Sun et al., 2012). This antigenic drift might have led to immunization failure and may explain the current prevalence of the H9N2 influenza virus in China.

**Figure 1:**
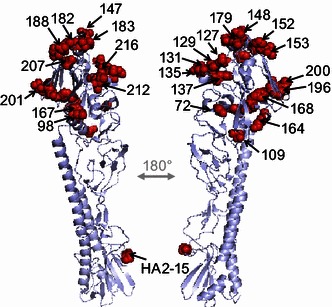
**Localization of amino acids related to the antigenicity of H9N2 influenza virus on the three-dimensional map of A/Swine/Hong Kong/9/98**. PDB ID is 1JSD. All positions are shown with H9 numbering

The identification of antigenic sites of H9 is important for monitoring antigenic variants and developing effective vaccines. Although the crystal structure of H9 has been determined (Ha et al., 2001), detailed H9 antigenic epitopes have not been elucidated. The identification of amino acids in H9 antigenic sites revealed different distribution of antigenic areas among other subtypes. Unlike the subtypes analyzed so far, H9 hemagglutinin does not contain an antigenic site corresponding to site A in H3 hemagglutinin (Kaverin et al., 2004). Multiple amino acid positions in HA protein related to the antigenicity of H9N2 viruses were identified, most of which located in the distal head of the HA trimer (see Fig. 1) (Kaverin et al., 2004; Okamatsu et al., 2008; Wan et al., 2014).

## INTERSPECIES TRANSMISSION OF H9N2 VIRUS IN CHINA

Occasional cases of H9N2 infecting humans have been reported in Southern China and Hong Kong (see Table 1), but there has been no evidence for human-to-human transmission (Uyeki et al., 2002). Patients presented with a mild and typical human flu-like illness that can easily be overlooked (Butt et al., 2005; Gou et al., 2000). In fact, serologic surveillance revealed that the number of humans infected by H9N2 virus were much higher than that of the confirmed cases. Poultry workers are considered to be at high risk of infection with avian influenza due to their frequent exposure to chickens. About 2.3%–4.6% of poultry workers had antibodies against H9 (Huang et al., 2013; Yu et al., 2013).

**Table 1:** Natural H9N2 influenza cases in humans in China

Year	Location	Patient characteristics	Clinical signs	Virus	Exposure to live poultry	References
1998	Guangdong province	14-Year-old male	Acute respiratory infection	A/Shaoguan/402/98	Lived with chickens in the same house	Guo et al. 1999
		75-Year-old male	Acute respiratory infection	A/Shaoguan/402/98	There is a farmer’s market near his home	
		4-Year-old male	Acute respiratory infection	A/Shantou/217/98	Unknown	
		1-Year-old female	Acute respiratory infection	A/Shantou/239/98	Unknown	
		36-Year-old female	Acute respiratory infection	A/Shantou/252/98	Yes	
1999	Guangdong province	22-Month-old female	Fever (38°C), cough	A/Guangzhou/33/99 (BJ/94- lineage)	No	Guo et al. 2002; Guo 2000
1999	Hong Kong	13-Month-old female	Fever (39.5°C), poor appetite, vomiting, inflamed oropharynx	A/Hong Kong/1073/99, A/Hong Kong/1074/99 (G1-lineage)	One patient was possibly exposed to chickens in the weeks prior to illness	Saito et al. 2001; Subbarao and Katz 2000; Uyeki et al. 2002
		4-Year-old female	Fever (38.9°C), malaise, sore throat, headache, vomiting, abdominal pain, diarrhea, inflamed oropharynx			
2003	Hong Kong	5-Year-old man	Mild fever, cough, and mild dehydration with dried lips and decreased skin turgor	A/Hong Kong/2108/03 (BJ/94-lineage)	No	Butt et al. 2005
2007	Hong Kong	9-Month-old female	Mild upper respiratory infection	Unknown	Unknown	http://www.chp.gov.hk
2008	Shenzhen SAR	Female	Cough and vomiting	A/Hong Kong/3239/2008	Unknown	http://www.chp.gov.hk
2013	Shenzhen SAR	86-Year-old man	Cold and cough	Unknown	No	http://www.chp.gov.hk
2013	Hunan province	7-Year-old man	Fever and rhinorrhea	Unknown	Yes	http://www.21hospital.com

H9N2 influenza viruses in poultry also transmitted to pigs, generating variants with novel antigenic and genetic characteristics (Cong et al., 2007; Peiris et al., 2001). Recently, an avian origin H9N2 influenza virus was isolated from dogs in Southern China, and the positive rates of serums from dogs in Southern China were 20.21% in 2010, 28.98% in 2011, and 44.85% in 2012; suggesting the circulation of H9N2 virus among dogs (Sun et al., 2013a). The enlarged host range of the H9N2 influenza virus underscored the potential public threat.

## GENETIC AND BIOLOGICAL EVOLUTION OF THE H9N2 VIRUS IN CHINA

Phylogenetic analysis indicated that the HA and neuroaminidase (NA) genes of most H9N2 viruses in China belong to BJ/94-like and G1-like lineages. The hosts of BJ/94-like viruses included chickens, ducks, other minor poultry species, swine, and humans, while G1-like viruses were only isolated from minor poultry and humans in Southern China. Compared with the surface genes (HA and NA), the lineages of six internal genes show a greater diversity, indicating frequent reassortment of these genes. At least 35 genotypes, with some genotypes consisting of triple or quadruple reassortants, were found in poultry in China (Sun et al., 2010b; Xu et al., 2007).

The number of H9N2 viruses, possessing HA proteins with an alanine to serine substitution at the P5 cleavage site (position 316, by H9 numbering) and a three-amino-acid deletion in the NA stalk (positions 61–63) in China was increasing (Sun et al., 2010b; Xu et al., 2007) (see Table 3). The HA-A316S mutation increased the HA cleavage efficiency, and the short stalk NA improved the NA enzyme activity and the virus release from erythrocytes. Each mutation or a combination of these mutations increased the virulence of H9N2 virus in chickens and mice (Sun et al., 2013b). In China, increasingly H9N2 influenza viruses with HA-Q226L substitution were found. HA-226L allowed H9N2 viruses to preferentially infect nonciliated cells expressing mainly SA-ɑ-2,6-Gal receptors and to grow more efficiently in human airway epithelial cultures maintained at the air-liquid interface (Wan and Perez, 2007). Additionally, the substitutions in M2 protein associated with drug-resistance were observed in Chinese H9N2 viruses since 1998 (Sun et al., 2010b).

It is noteworthy that H9N2 viruses present are with increasing adaption to both chickens and mammalians. Our chicken experiment showed that the titers of the H9N2 viruses in the tracheas of the inoculated and contact birds isolated after 2003 were hundred to thousand times higher than those in the earlier isolates (Sun et al., 2010b). According to an experiment performed by Li et al., H9N2 viruses isolated before 2000 could not be recovered from any of the organs of inoculated mice, and the mice stayed healthy and kept gaining weight. By contrast, those isolated between 2000 and 2002 induced disease signs and weight loss (up to 20% weight loss) (Li et al., 2005). Some H9N2 viruses isolated between 2007 and 2009 were reported to be highly lethal to mice and able to spread systemically, comparable with the highly pathogenic avian influenza virus (HPAI) (Bi et al., 2010; Deng et al., 2010). However, few reports similar to those of Bi et al. (2010) and Deng et al. (2010) have been published. The possibility of contamination of the H9N2 viruses isolated in the above-mentioned two studies with HPAI or bacteria cannot be excluded.

## VIRULENCE, TRANSMISSIBILITY, AND THE MOLECULAR MECHANISM OF THE H9N2 VIRUS IN ANIMAL MODELS

H9N2 influenza virus replicated efficiently in several animal models, including chickens, quails, BALB/c mice (Li et al., 2005), guinea pigs (Sun et al., 2010a), and ferrets (Ku et al., 2014) (see Table 2)—these animals are natural host species of the H9N2 virus. Quail were, without showing any signs of disease, more susceptible to the G1-lineage of the H9N2 virus than turkeys; while turkeys were associated with worse clinical conditions after being infected with G1-like H9N2 viruses (Bonfante et al., 2013). Some isolates could transmit among guinea pigs and ferrets via direct contact, but not by respiratory droplets (Lv et al., 2012; Wan et al., 2008) (see Table 2). Rhesus macaque, a non-human primate model, infected by the H9N2 virus, presented with biphasic fever and viral pneumonia, and the virus replicated in both their upper and lower respiratory tracts (Zhang et al., 2013a). The dogs and cats were also susceptible to avian H9N2 virus via the upper respiratory tract, and the virus could transmit between cats but not between dogs (Zhang et al., 2013b).

**Table 2:** Infection and transmission of H9 influenza viruses in mammalian models

Model	Type of virus	Infection	Direct contact transmission	Aerosol transmission	Reference
Mouse	H9N2	+	ND	ND	Sun et al. 2013b
Ferret	H9N2	+	+	−	Wan et al. 2008
	H9N2(HANA)-H3N2 reassortant	+	+	−	Sorrell et al. 2009
	H9N2(HANA)-H3N2 reassortant adaptive variants	+	+	+	Sorrell et al. 2009
	H9N2(HA)-H1N1 reassortant	+	+	−	Kimble et al. 2014; Kimble et al. 2011
	H9N2(HA)-H1N1 reassortant adaptive variants	+	+	+	Kimble et al. 2014; Kimble et al. 2011
	H9N2(HANA)-H1N1 reassortant	+	+	+	Kimble et al. 2011
Guinea pig	H9N2	+	+	−	Lv et al. 2012
Rhesus macaures	H9N2	+	ND	ND	Zhang et al. 2013a
Cat	H9N2	+	+	ND	Zhang et al. 2013b
Dog	H9N2	+	−	ND	Zhang et al. 2013b

ND, not done

The H9N2 virus with PB2-D253N and PB2-Q591K had greater polymerase activity, induced more tumor necrosis factor alpha (TNF-ɑ) in human macrophages, replicated better in differentiated normal human bronchial epithelial (NHBE) cells, and were more pathogenic in mice (Mok et al., 2011). Serial passages of H9N2 influenza virus in the animal models suggested that threat of H9N2 virus for public health might be increasing. An H9N2 virus could replicate and transmit in quail, but replicated poorly and did not transmit in chickens (Hossain et al., 2008). After 23 serial passages in quail, followed by 10 serial passages in chickens; this virus became very efficient at replicating and transmitting in quail and chickens. Moreover, the passaged viruses, unlike the parent virus, were able to readily infect mice and showed faster replication kinetics in tissue culture. These results are in agreement with the notion that adaptation of H9 viruses to land-based birds can lead to strains with expanded host range. A mouse-adapted H9N2 virus, generated by serial lung-to-lung passage, gained improved growth characteristics in mammalian cells, extended tissue tropism in mice, and was lethal for mice (Wu et al., 2009). We found that the combination of M147L and E627K on PB2 protein was responsible for the enhanced viral replication ability and increased virulence in mice of a mouse-adapted H9N2 virus (Wang et al., 2012b) (see Table 3).

**Table 3:** Determinants of virulence, transmissibility, or adaptation to mammals in H9N2 influenza virus

Key residues	Function	Reference
HA1-T189A, HA2-G192R	Necessary for respiratory droplet transmission between ferrets	Sorrell et al. 2009
HA-Q226L	Changes receptor binding specificity from SAɑ2,3Gal to SAɑ2,6Gal and increases replication in human airway epithelial cultures	Wan and Perez 2007
HA-A316S	Increases virulence in chickens and mice	Sun et al. 2013b
3-Amino-acid deletion in NA (positions 61–63)	Increases virulence in chickens and mice	Sun et al. 2013b
PB2-D253N, PB2-Q591K	Increase polymerase activity, replication in NHBE cells, and pathogenicity in mice, induce more TNF-ɑ in human macrophages	Mok et al. 2011
PB2-M147L, PB2-E627K	Increase pathogenicity in mice	Wang et al. 2012b

Because of the wide host range of H9N2, the possibility of reassortment between H9N2 and other influenza viruses in the future cannot be excluded. High genetic compatibility was found between H9N2 and H1N1/09 influenza viruses (Sun et al., 2011), and reassortants carrying the HA gene alone or in combination with the NA gene of an H9N2 virus in the H1N1/09 genetic background were respiratory droplet-transmissible between ferrets (Kimble et al., 2014; Kimble et al., 2011). Sorrell et al. found that following adaptation in the ferret, a reassortant virus carrying the surface proteins of an avian H9N2 into a human H3N2 backbone could gain respiratory transmissibility, and the adaptive changes in HA1-T189A and HA2-G192R were demonstrated to be necessary for respiratory droplet transmission (Sorrell et al., 2009) (see Table 3).

## H9N2 AS GENE DONOR TO NOVEL INFLUENZA VIRUSES

**Figure 2:**
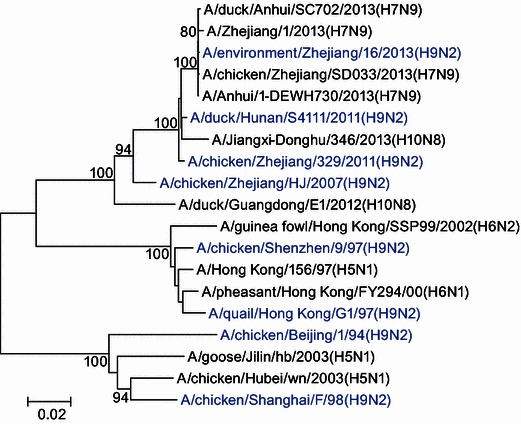
**Phylogenetic tree of the PB2 gene of representative influenza viruses which are genetically related to H9N2 viruses in China**. The unrooted phylogenetic tree was generated with MEGA 5.0, using the distance based neighbor-joining method. The reliability of the tree was assessed by bootstrap analysis with 1000 replications. The analysis was based on nucleotides 10–2241. H9N2 viruses are shown in blue

H9N2 influenza virus has been recognized to reassort with multiple other subtypes, including H6N1, H6N2, and H5N1 viruses (Shi et al., 2014; Sun et al., 2010b) (see Fig. 2). The G1-like H9N2 viruses were the likely donors of the six internal genes of the H5N1 viruses causing the bird flu outbreak which transmitted to humans in Hong Kong in 1997 (Guan et al., 1999), and the BJ/94-like H9N2 viruses provided the six internal genes to H7N9 and H10N8 viruses which emerged in humans in China since 2013 (Cui et al., 2014; Liu et al., 2014). Moreover, H7N9 influenza viruses continued to reassort with circulating H9N2 viruses, resulting in multiple genotypes of H7N9 viruses (Wu et al., 2013).

## THE FUTURE

The reassortant viruses derived from the H9N2 virus posed a great threat to public health. Compared with early isolates, recent H9N2 influenza viruses are much easier to infect mammals. The contribution of H9N2 genes, especially ribonucleoprotein (RNP) genes, to the infection in human needs to be determined. Additionally, to prevent and control avian influenza, veterinary biosecurity on farms need to be increased, rather than vaccine application alone, and live poultry markets should be shut down.
